# Gilteritinib-Induced Hypopituitarism: A Case Report

**DOI:** 10.7759/cureus.70401

**Published:** 2024-09-28

**Authors:** Yuri Hori, Yosuke Okada, Satomi Sonoda, Keiichi Torimoto, Yoshiya Tanaka

**Affiliations:** 1 Internal Medicine, School of Medicine, University of Occupational and Environmental Health, Kitakyushu, JPN

**Keywords:** acute myeloid leukemia, drug withdrawal, gilteritinib, hypopituitarism, pituitary hormone

## Abstract

Gilteritinib is a tyrosine kinase inhibitor (TKI) that treats acute myeloid leukemia (AML) by inhibiting FMS-like tyrosine kinase 3 (FLT3). This is a report on hypopituitarism induced by gilteritinib and its resolution following withdrawal. A 54-year-old woman was treated with gilteritinib for AML. She subsequently developed general fatigue. Blood tests showed low levels of anterior pituitary hormone. After 10 months of gilteritinib withdrawal, the levels of anterior pituitary hormones returned to normal values. When nonspecific symptoms such as fatigue in patients treated with gilteritinib are coupled with electrolyte abnormalities, a close checkup for hypopituitarism is recommended.

## Introduction

Acute myeloid leukemia (AML) is a disease caused by abnormal differentiation of myeloid cells, and it is known to be triggered by various genetic mutations. The prognosis of AML is also determined by the type of genetic mutation, and AML with mutations in the FMS-like tyrosine kinase 3 (FLT3) gene, which is a receptor-type tyrosine kinase, is known to have a poor prognosis [1].

Tyrosine kinase inhibitors (TKIs) are molecularly targeted drugs that specifically inhibit tyrosine kinases in cells and suppress intracellular signaling, with a resultant antitumor effect. Gilteritinib is a TKI that suppresses the growth of AML cells by inhibiting FLT3 and its downstream phosphorylation in patients with Internal tandem duplication (ITD) or tyrosine kinase domain (TKD) mutations [2]. ITD is a mutation that occurs when a specific region within the FLT3 gene is duplicated and repeated. The abnormal protein synthesized by the mutation overactivates the FLT3 receptor and promotes the abnormal proliferation of leukemic cells. TKD mutations are mutations in the FLT3 gene that occur in a region of the FLT3 receptor called the "tyrosine kinase domain." The TKD plays a crucial role in intracellular signal transmission, and mutations in this region cause persistent activation of the FLT3 receptor. TKIs are effective against these FLT3 gene mutations. This property makes it a promising drug that can potentially improve the prognosis of AML [3].

With regard to endocrine disorders associated with the use of gilteritinib, one global phase III study (ADMIRAL study) described a case of secondary hypoadrenocorticism, and another case of adrenal bleeding was described in a domestic post-marketing report, while a phase 1/2 study (CHRYSALIS study) and a domestic post-marketing survey described a single patient each who developed gilteritinib-related hypothyroidism [4-6]. In this way, reports of endocrine disorders following gilteritinib administration are scarce, and to our knowledge, there are no reports of gilteritinib-related hypopituitarism. Additionally, there are no existing reports detailing the progression of endocrine disorders. In this case, hypopituitarism developed after gilteritinib administration, and we were able to observe the long-term course following discontinuation of the drug. Therefore, we report here a rare case of spontaneous resolution of hypopituitarism after discontinuation of gilteritinib treatment.

Unknown endocrine disorders may occur after the administration of novel molecularly targeted therapies. Early detection and treatment of endocrine disorders would avoid interruption of treatment of malignant tumors and might prolong the life expectancy of patients. Therefore, it is important to report endocrine disorders that occur after the administration of new molecularly targeted therapies and their pathogenesis.

This article was previously presented as a poster presentation at the 95th Annual Congress of the Japan Endocrine Society on June 2-4, 2022.

## Case presentation

The patient was a 54-year-old woman (height: 152 cm; weight: 42 kg; BMI: 18.2 kg/m^2^). In July 2020, she was diagnosed with AML M2 and received idarubicin and cytarabine as remission-induction therapy, followed by complete remission (CR) in September of the same year. During the remission induction therapy, hydrocortisone was administered intravenously at 200 mg/day for five days from the end of September to early October, for four days in November, and for four days in December, with a total of 14 days of administration in the year 2020. The patient achieved CR through remission induction therapy, and after undergoing consolidation therapy, an FLT3 mutation was identified, leading to the decision to start gilteritinib treatment. Gilteritinib was administered once daily for a total of 64 days from February to April of the year 2021. Gilteritinib was discontinued after two months of treatment due to two side effects: fatigue and hyperglycemia. Five weeks after the withdrawal of gilteritinib, she was treated with intravitreal triamcinolone for leukemic retinopathy, ophthalmalgia, and conjunctivitis. One week later, she showed worsening general malaise, loss of appetite, and weight loss. Two months after gilteritinib withdrawal, body weight had decreased by 5 kg within a period of two weeks. The medical history was negative for thirst, polyuria, and polydipsia. Blood tests showed Na of 128 mEq/L, adrenocorticotropic hormone (ACTH) of 5.5 pg/mL, and cortisol of 0.7 μg/dL, suggesting secondary hypoadrenocorticism on 24 June 2021. She was admitted to our department for further management.

**Figure 1:**
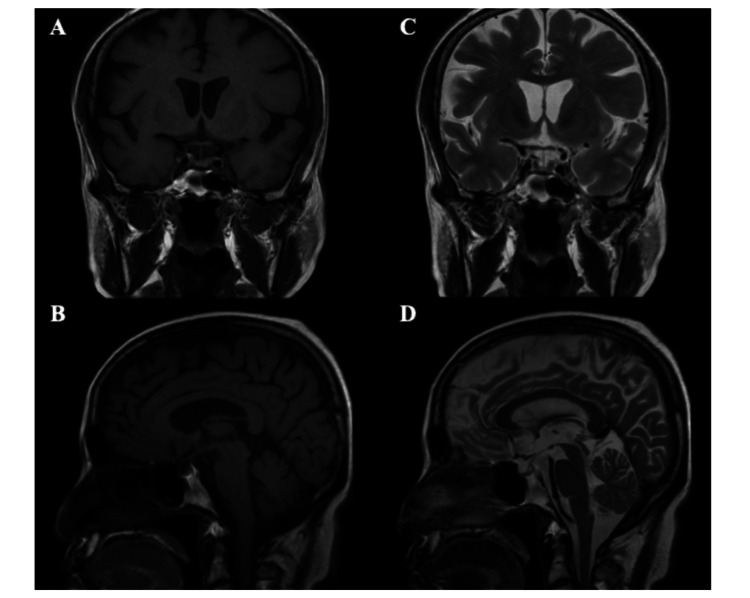
The pituitary MRI images The patient’s pituitary gland T1WI coronal (A) and axial (B) images and T2WI coronal (C) and axial (D) images are shown. No organic lesions were observed in the pituitary region. T1WI, T1-weighted images; T2WI, T2-weighted images

**Figure 2:**
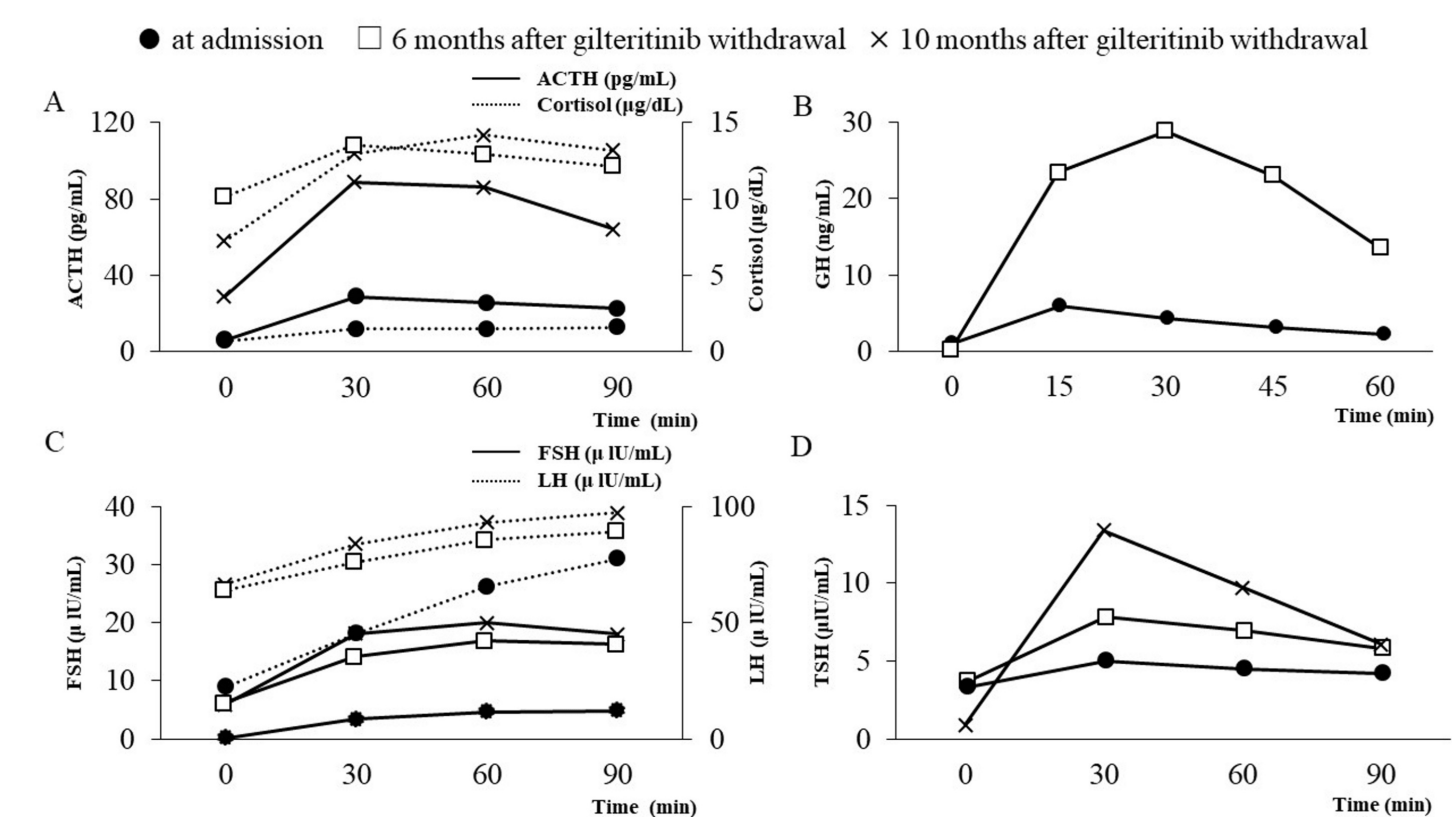
Results of load test (A) Results of the CRH load test. The CRH load test showed a normal ACTH response and low cortisol response at six and 10 months of withdrawal. The peak levels of both ACTH and cortisol improved after 10 months of withdrawal compared with those at admission. (B) Results of the GHRP-2 test. The GHRP-2 loading test showed low response, with a GH peak value of <9 ng/mL on admission. GH's response recovered after six months of withdrawal. (C) Results of the LHRH load test. The LH and FSH showed delayed response on admission. Both LH and FSH responses improved after six and 10 months of withdrawal. (D) Results of the TRH load test. The low response was evident in the TRH loading test on admission. TSH response recovered after six and 10 months of withdrawal. GH, growth hormone; FSH, follicle-stimulating hormone; LH, luteinizing hormone; CRH, corticotrophin-releasing hormone; ACTH, adrenocorticotropic hormone; GHRP-2, growth hormone releasing peptide-2; LHRH, luteinizing hormone-releasing hormone

Laboratory tests on admission (Table 1) showed normal Na, however, showed low levels of ACTH, cortisol, thyroid-stimulating hormone (TSH), insulin-like growth factor 1 receptor (IGF-1), luteinizing hormone (LH), follicle-stimulating hormone (FSH), Estradiol (E2), and progesterone. No structural changes were evident in the hypothalamus or pituitary gland on contrast-enhanced pituitary magnetic MRI (Figure 1). Urine output was 2200 mL/day during hospitalization. Further tests were performed to examine anterior pituitary function. Based on the above findings and the results of the corticotrophin-releasing hormone (CRH), growth hormone-releasing peptide 2 (GHRP-2), luteinizing hormone-releasing hormone (LHRH), and thyrotropin-releasing hormone (TRH) loading tests are shown in Figures 2A-2D. The CRH loading tests (Figure 2A) showed normal response of ACTH, low response of cortisol, and decreased secretion of multiple anterior pituitary hormones, leading to the diagnosis of c central adrenal insufficiency. The GHRP-2 loading test (Figure 2B) showed a low response, with a growth hormone (GH) peak value of <9 ng/mL. The LH and FSH showed delayed response (Figure 2C), respectively, and low response was evident in the TRH loading test (Figure 2D). The patient was diagnosed with hypopituitarism, which encompasses central adrenal insufficiency, central hypothyroidism, central hypogonadism, and growth hormone deficiency.

**Table 1:** Laboratory findings on admission AST, aspartate aminotransferase; ALT, alanine aminotransferase; BUN, blood urea nitrogen; CRP, C-reactive protein; IgG4, immunoglobulin G4; TRAb, thyroid stimulating hormone receptor antibody; TgAb, anti-thyroglobulin antibody; TPOAb, anti-thyroid peroxidase antibody

Measurement	Results	Reference range
Leukocyte count	4100/mL	3300-8600
Neutrophil	80.5%	40.0-75.0
Lymphocyte	12.1%	18.0-49.0
Eosinophil	0%	0.0-8.0
Basophil	0.2%	0.0-2.0
Monocytes	7.2%	2.0-10.0
Erythrocyte count	308×10^4 ^/μL	386-492×10^4^
Hemoglobin	11.8 g/dL	11.6-14.8
Platelet count	10.1×10^4 ^/μL	15.8-34.8
Total protein	5.6 g/dL	6.6-8.1
Albumin	3.7 g/dL	4.1-5.1
AST	33 IU/L	13-30
ALT	76 IU/L	7-23
BUN	16 mg/dL	8-20
Creatinine	0.35 mg/dL	0.46-0.79
Triglycerides	236 mg/dL	50-149
LDL-cholesterol	109 mg/dL	70-139
HDL-cholesterol	67 mg/dL	40-96
Na	139 mEq/L	138-145
K	4.1 mEq/L	3.6-4.8
Cl	103 mEq/L	101-108
Ca	9.1 mg/dL	8.5-10.2
CRP	0.04 mg/dL	0.00-0.14
IgG4	15.8 mg/dL	11-121
TRAb	1.0 U/mL	<2.0
TgAb	10 U/mL	<28
TPOAb	11 U/mL	<16

Based on the diagnosis, the patient was treated for hypoadrenocorticism with oral hydrocortisone at 15 mg/day. Replacement therapy for the severe growth hormone deficiency was not indicated since the patient was undergoing AML treatment. Replacement therapy was also not indicated for gonadotropin deficiency and central hypothyroidism since the patient was menopausal and FT4 levels were within the normal ranges. 

Table 2 shows the basal values of the anterior pituitary hormones at six months after gilteritinib withdrawal and at 10 months after gilteritinib withdrawal. The data show that the levels of various anterior pituitary hormones returned to normal values after six months of withdrawal and remained stable and did not change after 10 months of discontinuation. The CRH load test (Figure 1A) showed a normal ACTH response and low cortisol response at six and 10 months of withdrawal. The peak levels of both ACTH and cortisol improved after 10 months of withdrawal compared with those at admission. The GHRP-2 load test (Figure 1B) showed a normal GH response after six months of withdrawal. In the LHRH load test (Figure 1C), both LH and FSH levels improved after six and 10 months of withdrawal. These results indicated recovery from severe growth hormone deficiency after six months of gilteritinib withdrawal, together with recovery from hypopituitarism after 10 months of gilteritinib withdrawal.

**Table 2:** Changes in hormonal assay tests after gilteritinib withdrawal ACTH, adrenocorticotropic hormone; GH, growth hormone; IGF-1, insulin-like growth factor-1; TSH, thyroid stimulating hormone; LH, luteinizing hormone; FSH, follicle-stimulating hormone; E2, estradiol

Parameter	Time after gilteritinib withdrawal (months)			Reference range
	2	6	10	
ACTH (pg/mL)	3.7	10	106.0	7.2-63.3
Cortisol (μg/dL)	0.7	0.8	13.7	7.07-19.6
GH (ng/mL)	0.42	16.7	4.93	0.13-9.8
IGF-1 (ng/mL)	49	226	167	76-211
TSH (μIU/mL)	0.19	1.80	0.65	0.35-4.94
Free T3 (pg/mL)	1.16	-	-	2.47-4.34
Free T4 (ng/dL)	1.23	1.32	1.06	0.70-1.48
LH (μIU/mL)	1.3	15.7	15.0	5.16-61.99
FSH (μIU/mL)	13.5	28.6	26.1	26.72-133.41
E2 (pg/mL)	<5.0	<5.0	<5.0	<39.0
Progesterone (ng/dL)	<0.1	<0.1	0.2	<0.33
Prolactin (ng/mL)	16.9	24.5	23.8	3.12-15.39

The patient continued to show hematologic remission with no dysplasia on bone marrow imaging at four months after gilteritinib administration and after discontinuation, suggesting its therapeutic effectiveness in AML. After 10 months of gilteritinib withdrawal, bone marrow transplantation became necessary for relapse of AML. Furthermore, the cortisol dose was increased based on high-stress levels during the subsequent development of organizing pneumonia, in addition to the continuation of hydrocortisone replacement therapy. The potential of hydrocortisone dose reduction or discontinuation will be assessed in the future upon the expected improvement in the clinical condition.

## Discussion

Gilteritinib is a molecular targeting agent for AML [2]. In patients with either FLT3-ITD or FLT3-TKD mutations, which are considered poor prognostic factors for AML, gilteritinib exhibits antitumor activity by inhibiting tyrosine kinases against FLT3 mutations. This compound has been reported to have a composite CR rate of 37.2% and a response rate of 48.7%, making it an important drug that can potentially improve the prognosis of AML in the future [3].

Regarding endocrine disorders associated with the use of gilteritinib, one case of secondary hypoadrenocorticism was reported overseas in a global phase III study (ADMIRAL study), one case of adrenal bleeding in a domestic post-marketing report, and hypothyroidism was reported in an overseas phase 1/2 study (CHRYSALIS study) and a domestic post-marketing survey [4-6]. However, there have been no reports of hypopituitarism nor endocrinological studies that confirmed recovery after drug discontinuation. 

Although the mechanism of gilteritinib-induced endocrine grand disruption remains elusive, in our patient, FLT3 inhibitors caused secondary hypoadrenocorticism and central hypothyroidism, and over time after discontinuation, symptoms improved and hormone levels recovered.

With respect to TKIs and endocrine dysfunction, it is known that thyroid and parathyroid dysfunction are associated with TKIs. The mechanism of these abnormalities is thought to be related to the fact that TSH and parathyroid hormone (PTH) receptors are G-protein-coupled receptors (GPCR). Recent studies have reported that the signal transduction pathways of both tyrosine kinases and GPCR signaling pathways converge in the mitogen-activated protein kinase (MAPK) cascade and that cross-talk between the two receptors occurs in both at the protein-protein interaction level and downstream of their respective signal transduction cascades, suggesting a possible mechanism for the endocrine-related side effects of TKIs [7,8]. Both CRH and TRH receptors are GPCR and may be triggered by a similar mechanism.

Indeed, it has been reported that Lapatinib, a TKI for corticotroph tumor cells, reduces ACTH production by decreasing the mRNA expression level of proopiomelanocortin (POMC) [9].

We recognized that the appearance of the clinical features of adrenal insufficiency, despite the withdrawal of gilteritinib, was related to the highly stressful intravitreal triamcinolone treatment for leukemic retinopathy. To date, the mechanism of resolution in previously reported gilteritinib-induced endocrine disturbances is unclear, and whether this is a reversible adverse effect that improves after the discontinuation of gilteritinib remains unknown. In our case, the low levels of ACTH, GH, TSH, and LH/FSH observed after gilteritinib administration were followed by normalization of the GH secretory disturbance and improvement in TSH secretory kinetics at six months after withdrawal of gilteritinib. These post-withdrawal endocrine improvements suggest that gilteritinib endocrine disruption is reversible. Current therapeutic protocols advocate the use of gilteritinib continuously unless AML relapses or progresses or serious adverse effects occur [4]. It is possible that the reversible nature of endocrine disturbances observed in our case was related to the short duration of gilteritinib administration.

Gilteritinib is expected to play an important role in the treatment of AML and its use is expected to increase in the future [10]. Among the adverse reactions, nausea (15%), fatigue (24%), headache (11%), and hyponatremia (21%) have been reported [11]. In our case, the presenting symptoms of hypopituitarism, such as general malaise, poor appetite, and hyponatremia, triggered the identification of gilteritinib-induced disruptions.

The limitation of the current report is the lack of endocrinological testing related to pituitary function prior to gilteritinib administration. In general, endocrinological testing is not mandatory prior to gilteritinib administration and was not performed on the patient in the present report. However, clinical symptoms suggestive of hypopituitarism, such as general malaise, anorexia, and hyponatremia, did not occur prior to gilteritinib administration, so we do not believe that the patient could have developed hypopituitarism prior to gilteritinib administration.

Although various gilteritinib-related adverse reactions have been reported, this is the first detailed report of hypopituitarism associated with gilteritinib and the subsequent endocrinological improvement after its discontinuation, as confirmed by repeated stress tests. Based on this case, we recommend the need to rule out hypopituitarism in AML patients who report the appearance of vague symptoms, such as general malaise and loss of appetite, after gilteritinib administration.

## Conclusions

In conclusion, we encountered a patient who developed hypopituitarism after the use of gilteritinib and showed significant improvement in pituitary function after discontinuation of the drug. Until now, due to the lack of case reports like this one, when AML patients treated with gilteritinib presented with non-specific symptoms such as general malaise, poor appetite, nausea, and vomiting, discontinuation of gilteritinib might have been the only option, and if the symptoms improved upon discontinuation, further examination might not have been conducted. Through this report, we have raised the important awareness that such symptoms may indicate hypopituitarism, and in the future, proactive endocrinological screening to exclude hypopituitarism should be performed. Furthermore, pituitary dysfunction caused by gilteritinib may be reversible, and pituitary function should be re-evaluated after discontinuation of the drug. However, these mechanisms have not been validated clinically and further studies are needed to determine the relationship between response to gilteritinib and duration of treatment and extent of endocrine disturbances.
